# A Fast Superpixel Segmentation Algorithm for PolSAR Images Based on Edge Refinement and Revised Wishart Distance

**DOI:** 10.3390/s16101687

**Published:** 2016-10-13

**Authors:** Yue Zhang, Huanxin Zou, Tiancheng Luo, Xianxiang Qin, Shilin Zhou, Kefeng Ji

**Affiliations:** 1 College of Electronic Science and Engineering, National University of Defense Technology, Changsha 410073, China; yue1554415@163.com (Y.Z.); 15616045932@163.com (T.L.); slzhou@nudt.edu.cn (S.Z.); jikefeng@nudt.edu.cn (K.J.); 2 School of Information and Navigation, Air Force Engineering University, Xi’an 710077, China; qxxzhijia@126.com

**Keywords:** superpixel segmentation, edge refinement, revised Wishart distance, unstable pixels, PolSAR images

## Abstract

The superpixel segmentation algorithm, as a preprocessing technique, should show good performance in fast segmentation speed, accurate boundary adherence and homogeneous regularity. A fast superpixel segmentation algorithm by iterative edge refinement (IER) works well on optical images. However, it may generate poor superpixels for Polarimetric synthetic aperture radar (PolSAR) images due to the influence of strong speckle noise and many small-sized or slim regions. To solve these problems, we utilized a fast revised Wishart distance instead of Euclidean distance in the local relabeling of unstable pixels, and initialized unstable pixels as all the pixels substituted for the initial grid edge pixels in the initialization step. Then, postprocessing with the dissimilarity measure is employed to remove the generated small isolated regions as well as to preserve strong point targets. Finally, the superiority of the proposed algorithm is validated with extensive experiments on four simulated and two real-world PolSAR images from Experimental Synthetic Aperture Radar (ESAR) and Airborne Synthetic Aperture Radar (AirSAR) data sets, which demonstrate that the proposed method shows better performance with respect to several commonly used evaluation measures, even with about nine times higher computational efficiency, as well as fine boundary adherence and strong point targets preservation, compared with three state-of-the-art methods.

## 1. Introduction

Polarimetric synthetic aperture radar (PolSAR) is popular and attractive due to its advantage of providing richer information with four channels HH, HV, VH and VV, where HH, HV, VH and VV denote the polarization models for horizontal transmit and horizontal receive (HH), horizontal transmit and vertical receive (HV), vertical transmit and horizontal receive (VH), and vertical transmit and vertical receive (VV), respectively. Many researches have been developed on PolSAR images. Among them, PolSAR image classification [1,2,3,4,5] is a basic issue. There are basically two approaches for classification: (1) pixel-based classification; (2) region-based classification. Due to the presence of inherent speckle noise in PolSAR images, traditional pixel-based classification algorithms have some drawbacks [6], i.e., sensitivity to noise and more computation. Compared to the traditional pixel-based methods, region-based algorithms process images at region level instead of pixel level, making the information of regions more available with better anti-noise performance. However, additional region generation methods are generally required beforehand, e.g., superpixel algorithms.

Ren and Malik [7] firstly proposed the term superpixel—a group of pixels with similar color or other low-level features—in 2003. Superpixels have been commonly used for a wide range of applications, for example, as a preprocessing step for change detection [8,9], as mid-level cues used for tracking [10], or as a preprocessing step for classification [11,12,13]. So far, many superpixel segmentation algorithms have been proposed, which can be generally categorized into two classes: graph based methods and gradient ascent methods. Normalized Cuts (Ncut) [7], Entropy Rate Superpixels [14] and Pseudo Boolean Optimization (PB) [15] are the typical algorithms of graph based methods. For gradient ascent methods, there are QuickShift (QS) [16], Contour Relaxed Superpixels (CRS) [17] and Superpixels Extracted via Energy-Driven Sampling (SEEDS) [18]. Simple Linear Iterative Clustering (SLIC) [19], which is popular for its simplicity and good performance, can also be classified into the gradient ascent methods. All the algorithms mentioned above are originally proposed for optical images and have their own advantages for different images. However, when applied to PolSAR images, they may generate poor superpixels because of the serious influence of inherent speckle noise and many small-sized or slim regions in PolSAR images.

SLIC is attractive and contains three main steps: (1) initialization; (2) local k-means clustering; (3) postprocessing. Zou et al. [20] proposed an improved SLIC based on generalized Gamma distribution for SAR images. While the algorithm shows good performance in SAR data, it does not do so PolSAR data. Recently, many improved algorithms based on SLIC have been proposed for PolSAR images. Feng et al. [21] introduced an improvement over SLIC by utilizing the Wishart distance instead of color Euclidean distance. Although the algorithm yields better superpixels than SLIC, small isolated regions still exist and the computation efficiency is unsatisfactory. Another method based on SLIC, which employs a combined distance, including space distance, Wishart distance and edge gradient distance, was proposed by Xie et al. [9]. This method can preserve the edges very well, but with low computation efficiency. Qin et al. [22] proposed an improved SLIC based on the revised Wishart distance and improved postprocessing. The final superpixels are of good boundary adherence but with long running time and unsatisfactory regularity. Zhu et al. [23] introduced a fast superpixel segmentation algorithm by iterative edge refinement (IER), which is fast, homogeneous regular and boundary-adherent for optical images. However, IER cannot be directly applied to PolSAR images to yield superpixels due to the strong impacts of coherent speckle noise interference in PolSAR images.

To address these problems—inspired by the work in the literature [23]—an improved SLIC algorithm is proposed to generate fine superpixels for PolSAR images in this paper. Different from the IER originally designed for the superpixel generation of optical images, in the initialization step of our improved algorithm, we initialize unstable pixels as all the pixels in PolSAR images rather than the initial grid edge pixels in IER, so as to maintain all the potential edge pixels to eliminate the effects of small-sized or slim regions in PolSAR images. Moreover, when relabeling the unstable pixels in the local searching area, the revised Wishart distance, instead of Euclidean distance, is adopted as the data distance measure to reduce the impact of speckle noise. In the literature [24], a fast PolSAR image classification was proposed based on fast implementation of Wishart distance. Inspired by this, the revised Wishart distance is calculated by fast implementation to further increase computation efficiency. Finally, postprocessing with a dissimilarity measure is utilized to remove the generated small isolated regions and to preserve strong point targets at the same time. Four simulated and two real-world PolSAR images are exploited to evaluate the superior performance of the proposed method. The experimental results indicate that the proposed method may obtain better performance with respect to several commonly used evaluation measures with higher computational efficiency, as well as more accurate boundary adherence and strong point targets preservation, compared with three state-of-the-art methods.

## 2. IER for Optical Images

IER is a fast superpixel generation algorithm and shows fine performance when applied to optical images. In an image, unstable pixels [23] are those pixels whose labels are likely to change and should be checked in the next iteration. The definition of unstable pixels is given as follows:

(1) UP={p|nt(p)  ≠  nt(q)  and  nt(q)≠t(q),  q∈Nb(p)}

where p and q represent pixels in the image domain Ω , i.e., p,q∈Ω . Nb(p) is the neighborhood function and a 4-connected neighborhood is utilized in the experiments. t(i) represents the label of i and nt(i) represents the new label after one iteration, i=p,q .

The color-spatial combined distance [25] is used in IER to relabel the unstable pixels in the local clustering and LAB color space developed by International Commission of Illumination (CIELAB) is employed. The combined distance is defined by

(2) $$d_{c}\left(i,j\right)=\sqrt{{\left(l_{j}-l_{i}\right)}^{2}+{\left(a_{j}-a_{i}\right)}^{2}+{\left(b_{j}-b_{i}\right)}^{2}}$$

(3) $$d_{s}\left(i,j\right)=\sqrt{{\left(x_{j}-x_{i}\right)}^{2}+{\left(y_{j}-y_{i}\right)}^{2}}$$

(4) $$D\left(i,j\right)={\left(\frac{d_{c}\left(i,j\right)}{m}\right)}^{2}+{\left(\frac{d_{s}\left(i,j\right)}{S}\right)}^{2}$$

where the subscripts i and j represent the cluster center of the ith superpixel and the jth unstable pixel, respectively. S is the initial grid width and m is the compactness parameter. When m is a large value, it indicates that the spatial distance is more significant than the data distance, thus leading to more regular final superpixels. The main steps of the IER algorithm are shown in Algorithm 1:

**Algorithm 1.** IER for Optical Images.**given:** the initial grid width 
S
, color image 
I
**step 1.** (Initialization) Initialize the superpixels of 
I
 as regular grids with width 
S
. Create the models (mean color values and geometric centers) for all superpixels. Initialize the unstable pixels as the grid edge pixels. Set the iteration index *iter =* 0.**step 2.** (Local relabeling) If *iter ≥ itermax*, the maximum iteration times reached, or the unstable pixel set is empty, then the algorithm ends and goes to Step 4. If not, relabel all the unstable pixels by the color-spatial combined distance defined in Equation (2) in the local searching area 
2S×2S
.**step 3.** (Updating) Update the superpixel models and the unstable pixel set by its definition (1). Set *iter = iter +* 1 and return to Step 2.**step 4.** (Postprocessing) Remove the generated isolated regions with size smaller than 
$S_{\min}$
 by connected component analysis (CCA) based on the combined distance defined as Equation (2).**output:** superpixels

## 3. Proposed Superpixel Algorithm

IER is originally proposed to generate superpixels efficiently for optical images. However, when applied to PolSAR images, IER may generate poor superpixels due to the influence of inherent speckle noise and many small-sized or slim regions. In Section 3.1 we introduce the revised Wishart distance to replace the CIELAB Euclidean distance as the data distance measure. By doing so, the effect of speckle noise will be reduced significantly. Section 3.2 contains the initialization step of the proposed method for PolSAR images with many small-sized or slim regions. In Section 3.3, a postprocessing procedure based on the dissimilarity measure is introduced to remove the generated small regions as well as to preserve the strong point targets. Finally, the algorithm flow is given in four steps. Since our proposed method is motivated by the IER algorithm originally designed for the superpixel generation of optical images and is applied to PolSAR images in this paper, we call it Pol-IER for simplicity hereafter.

### 3.1. Revised Wishart Distance

#### 3.1.1. Traditional Revised Wishart Distance

The amplitude and phase of backscattering signals, which are in four combinations of linear, receive and transmit polarizations: HH, HV, VV, VH; usually contained in PolSAR data. Each pixel of a PolSAR image can be described by a complex matrix as follows:

(5) $$S=\left[\begin{array}{cc} S_{HH} & S_{HV}\\ S_{VH} & S_{VV} \end{array}\right]$$

where $S_{HV}=S_{VH}$ for a reciprocal medium. Generally, Pauli scattering vector $k_{p}$ can be used to represent the backscattering signals. It is defined by

(6) $$k_{p}=\frac{{\left[\begin{array}{ccc} S_{HH}+S_{VV} & S_{HH}-S_{VV} & 2S_{HV} \end{array}\right]}^{T}}{\sqrt{2}}$$

where the superscript T denotes the matrix transpose. For multilook PolSAR data, the polarimetric information can be described as the coherency matrix T , which can be generated from the outer product of $k_{p}$ . The expression can be denoted by

(7) $$T=\frac{1}{L}\sum_{p=1}^{L}k_{p}k_{p}^{*T}$$

where L is the number of looks and the superscript * denotes the complex conjugate. Let X=LT . Then X has the complex Wishart distribution [26], i.e., $X\sim W_{c}\left(L,q,\Sigma \right)$ , where $\Sigma =E\left(k_{p}k_{p}^{*T}\right)$ . Therefore, the probability density function (PDF) of X is given by

(8) $$p\left(X|q,\Sigma \right)=\frac{{\left|X\right|}^{L-q}}{\Gamma_{q}\left(L\right){\left|\Sigma \right|}^{L}}\exp\left(-\mathrm{Tr}\left[\Sigma^{-1}X\right]\right)$$

(9) $$\Gamma_{q}\left(L\right)=\pi^{q\left(q-1\right)/2}\prod_{j=1}^{q}\Gamma \left(L-j+1\right)$$

where Γ(n) is the Gamma function [27] and q is the dimension of $k_{p}$ , i.e., 3. |.| is the determinant of a matrix. Thus the PDF of T can be written as follows:

(10) $$p\left(T|q,\Sigma \right)=\frac{L^{Lq}{\left|T\right|}^{L-q}}{\Gamma_{q}\left(L\right){\left|\Sigma \right|}^{L}}\exp\left(-L\mathrm{Tr}\left[\Sigma^{-1}T\right]\right)$$

Let $\Sigma_{i}$ and $\Sigma_{j}$ be the center coherency matrices of the regions $R_{i}$ and $R_{j}$ , respectively. The hypothesis test [28] is

(11) $$\{\begin{array}{c} H_{0}:\Sigma_{i}=\Sigma_{j}\\ H_{1}:\Sigma_{i}\neq \Sigma_{j} \end{array}$$

Then the maximum-likelihood (ML) estimators of $\hat{\Sigma_{i}}$ and $\hat{\Sigma_{j}}$ are $\hat{\Sigma_{i}}=\left(\sum_{n=1}^{N_{i}}T_{n}\right)/N_{i}$ and $\hat{\Sigma_{j}}=\left(\sum_{n=1}^{N_{j}}T_{n}\right)/N_{j}$ , respectively. When $\Sigma_{j}$ is given for hypotheses $H_{0}$ and $H_{1}$ , the likelihood-ratio test statistic [22] is

(12) $$Q=\frac{{\left|\hat{\Sigma_{i}}\right|}^{LN_{i}}}{{\left|\hat{\Sigma_{j}}\right|}^{LN_{j}}}\exp\left\{-LN_{i}\left(\mathrm{Tr}\left({\hat{\Sigma_{j}}}^{-1}\hat{\Sigma_{i}}\right)-q\right)\right\}$$

Thus, the distance between $R_{i}$ and $R_{j}$ is the revised Wishart distance [26] defined by

(13) $$d_{RW}\left(R_{i},R_{j}\right)=\mathrm{In}\left(\frac{\left|\hat{\Sigma_{j}}\right|}{\left|\hat{\Sigma_{i}}\right|}\right)+\mathrm{Tr}\left(\hat{{\Sigma_{j}}^{-1}}\hat{\Sigma_{i}}\right)-q$$

So the revised Wishart distance between a pixel i with the coherency matrix $T_{i}$ and the jth cluster $R_{j}$ with the center coherency matrix $C_{j}=\left(\sum_{n=1}^{N_{j}}T_{n}\right)/N_{j}$ is

(14) $$d_{RW}\left(T_{i},R_{j}\right)=\mathrm{In}\left(\frac{\left|C_{j}\right|}{\left|T_{i}\right|}\right)+\mathrm{Tr}\left({C_{j}}^{-1}T_{i}\right)-q$$

In this paper, Equation (14) is utilized instead of $d_{c}\left(i,j\right)$ in Equation (4) to eliminate the influence of speckle noise in the step of local relabeling. In some literatures [21,29,30], Wishart distance defined as Equation (15) is employed to measure the distance between a pixel and a cluster. Our experiments on the simulated PolSAR data will illustrate that our algorithm with the revised Wishart distance outperforms the Wishart distance in terms of four commonly used criteria.

(15) $$d_{W}\left(T_{i},R_{j}\right)=\mathrm{In}\left(\left|C_{j}\right|\right)+\mathrm{Tr}\left({C_{j}}^{-1}T_{i}\right)$$

Therefore, the combined distance of the pixel *i* and the cluster $R_{j}$ used in this paper is

(16) $$D\left(i,R_{j}\right)={\left(\frac{d_{RW}\left(i,R_{j}\right)}{m}\right)}^{2}+{\left(\frac{d_{s}\left(i,R_{j}\right)}{S}\right)}^{2}$$

#### 3.1.2. Fast Implementation of Revised Wishart Distance

Since the revised Wishart distance will be calculated many times in the local relabeling step, a large amount of time will be spent in the computation of the distance. Therefore, a fast implementation [24] of the revised Wishart distance is employed in this paper to further increase the computation efficiency. As shown in Equations (14) and (15), the term $\mathrm{Tr}\left({C_{j}}^{-1}T_{i}\right)$ is traditionally calculated by multiplying ${C_{j}}^{-1}$ by the matrix $T_{i}$ firstly and then computing the trace of the matrix ${C_{j}}^{-1}T_{i}$ , which needs 27 multiplication operations and 20 addition operations with the obtained ${C_{j}}^{-1}$ . Let $\Gamma ={C_{j}}^{-1}T_{i}$ . It is known that both ${C_{j}}^{-1}$ and $T_{i}$ are 3×3 complex matrices, and so is Γ . To calculate the trace of Γ , we only need to summarize the diagonal elements of Γ . That is to say, it is redundant to calculate the matrix Γ and only necessary to obtain the diagonal elements. Therefore, the fast implementation of the revised Wishart distance is only to calculate the summary of the diagonal elements and leave out the redundant computation.

Based on the idea mentioned above, let σ=f(T) be a function that arranges all the elements of the matrix T into a vector. In this paper, $f(T)=[T_{11},T_{21},T_{31},T_{12},T_{22},T_{32},T_{13},T_{23},T_{33}]$ and it is noticed that $T_{ij}$ and $T_{ji}$ are conjugate symmetry for the coherency matrix T . Let $w_{j}=f\left({\left({C_{j}}^{-1}\right)}^{T}\right)$ and $t_{i}=f\left(T_{i}\right)$ , where ${\left(.\right)}^{T}$ means a matrix transpose without conjugation. Then it is easy to notice the fact that $\mathrm{Tr}\left({C_{j}}^{-1}T_{i}\right)={\left(w_{j}\right)}^{T}t_{i}$ , where both $w_{j}$ and $t_{i}$ are 9-dimensional vectors. Contrary to the high-computation of $\mathrm{Tr}\left({C_{j}}^{-1}T_{i}\right)$ , only nine multiplication operations and eight addition operations are needed to calculate ${\left(w_{j}\right)}^{T}t_{i}$ , which is only one-third of what $\mathrm{Tr}\left({C_{j}}^{-1}T_{i}\right)$ the traditional way needs. Therefore, the revised Wishart distance can be represented by

(17) $$d_{RW}\left(T_{i},R_{j}\right)=\mathrm{In}\left(\frac{\left|C_{j}\right|}{\left|T_{i}\right|}\right)+{\left(w_{j}\right)}^{T}t_{i}-q$$

### 3.2. Initialization of Unstable Pixels

**Figure 1:**
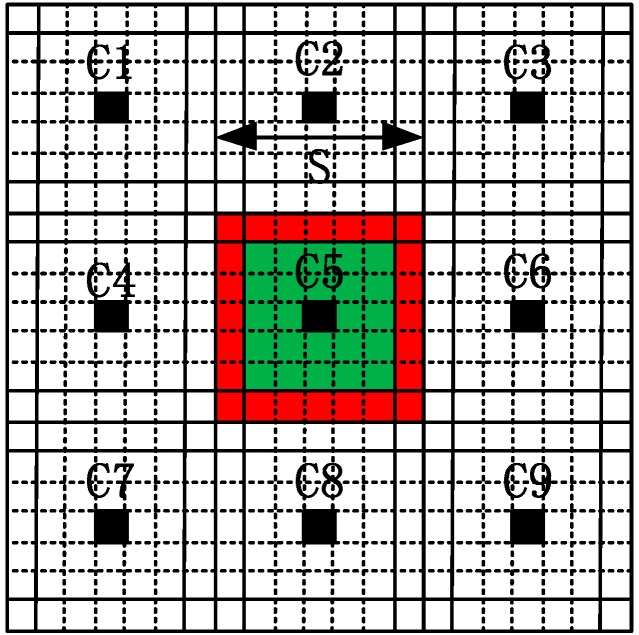
The sketch map of initialization of two methods. Ci indicates the *i*th cluster center, and S is the initial grid width. The pixels filled with black are the initial cluster centers. For the fifth cluster, the initial unstable pixels of the algorithm by iterative edge refinement (IER) are the red pixels, while the initial unstable pixels of the proposed method are the red, green and black pixels.

As shown in Figure 1, to generate superpixels using IER, an image is firstly divided into several grids with width S . For example, for the fifth cluster, the original center is set as C5 , i.e., the pixel filled with black. The IER algorithm initializes the unstable pixels as the regular grid edge pixels, for example, the red pixels in the edge of the fifth cluster. For optical images, this kind of initialization has a slight effect on the boundary adherence of the final superpixels, because optical images always contain several regions of regularity and homogeneity. By contrast, PolSAR images usually include many small regions, slim regions and strong point targets, which provide important information for image processing such as ship detection, change detection and classification of land use/land cover (LULC), and so on.

When applied to PolSAR images, IER with this kind of initialization may yield poor superpixels with many edges lost. In addition, the properties of good boundary adherence and homogeneous regularity should be obtained with as few superpixels as possible. This means that whether the final superpixels are good or not does not depend on the initial grid width. However, the final results of IER can be severely affected by the initial grid width. Since many real edge pixels may locate in the middle of the grid, when the original grid width is large relative to the image size, some true edges may be missed with this kind of initialization. Therefore, with a small grid width, IER may work well, and otherwise the opposite, which is not expected.

To fix the problems mainly caused by the initialization of IER for generating superpixels for PolSAR images, we initialize the unstable pixels as all the pixels in PolSAR images instead of the initial grid edge pixels. For the fifth cluster in Figure 1, our proposed initial unstable pixels consist of the red pixels, green pixels and the corresponding cluster center filled with black. By doing so, all the potential edges in PolSAR images will be maintained, resulting in accurate boundary adherence and thus accurate superpixels. Although the initialization step of our proposed method will slightly increase the running time at the first several iterations (Usually three or four iterations. See Section 4.1.2 for more details), we can ensure the preservation of all the potential edges, which is of significance for superpixels’ applications.

### 3.3. IER for PolSAR Data

Based on the above analysis, the IER for PolSAR data in this paper can be summarized as three steps to generate superpixels. Although some changes have been made based on the characteristics of PolSAR images, small isolated superpixels may still exist. To address this problem, the connected components algorithm (CCA), based on the combined distance in Equation (4) to remove small isolated regions by merging them into nearby large superpixels, is used in IER. Although it is easy to perform, it will lead to poor boundary adherence of superpixels for PolSAR images. That is because the generated small isolated regions may significantly differ from the adjacent superpixels.

In order to merge the generated small isolated regions as well as to preserve the strong point targets, a postpocessing procedure [21] based on a dissimilarity measure is introduced in this paper. The superpixels with the size smaller than the prespecified threshold $N_{th}=S^{2}/4$ are interesting. When the size of a superpixel is smaller than $N_{th}$ , the dissimilarities between this superpixel and the superpixels in its 8-connected neighborhood will be calculated. If the smallest dissimilarity value is smaller than a predefined threshold $G_{th}$ , we merge this superpixel into the neighbor with the smallest dissimilarity between them. If not, move to the next superpixel. The dissimilarity measure [14] between two superpixels $R_{i}$ and $R_{j}$ is defined as follows:

(18) $$G\left(R_{i},R_{j}\right)=\frac{1}{q}{\left\|\frac{C_{i}^{diag}-C_{j}^{diag}}{C_{i}^{diag}+C_{j}^{diag}}\right\|}_{1}$$

where $C^{diag}$ means the vector consisting of the diagonal elements of the center coherency matrix of a superpixel and ${\left\|.\right\|}_{1}$ denotes 1-norm of a matrix. Since the dissimilarity G belongs to [0, 1], $G_{th}$ is set as 0.3 in all the experiments throughout this paper.

The main steps of our improved IER for PolSAR data, namely Pol-IER, can be summarized in Algorithm 2.

**Algorithm 2.** Pol-IER for PolSAR images.**given:** the initial grid width 
S
, PolSAR image 
I
**step 1.** (Initialization) Initialize the superpixels of image 
I
 as regular grids with interval 
S
. Create the models (mean coherency matrices and geometric centers) for all the superpixels. Initialize the unstable pixels as all the pixels of the PolSAR image. Set the iteration index *iter* = 0.**step 2.** (Local relabeling) If *iter ≥ itermax*, the maximum iteration times reached, or the unstable point set is empty, then the algorithm ends and goes to Step 4. Or relabel all the unstable pixels by the combined distance defined in Equation (16). Assign each unstable pixel whose searching area is 
2S×2S
 to the closest cluster whose location is covered by the searching area.**step 3.** (Updating) Update the superpixel models and the unstable pixel set by the definition (1). Set *iter = iter* + 1 and return to Step2.**step 4.** (Postprocessing) Search the superpixels with the size smaller than 
$N_{th}$
. If the smallest dissimilarity calculated by Equation (18) is smaller than 
$G_{th}$
, merge it into the closest adjacent superpixel. If not, move to the next superpixel until each superpixel is checked.**output:** superpixels

## 4. Experiments and Discussion

To evaluate our proposed Pol-IER, extensive experiments on four simulated PolSAR images and two real-world images from Experimental Synthetic Aperture Radar (ESAR) and Airborne Synthetic Aperture Radar (AirSAR) were conducted. Section 4.1 introduces four commonly used criteria: comparison experiments of the Wishart distance, and the revised Wishart distance; evaluations on two initialization methods; and comparison experiments on four state-of-the-art algorithms (i.e., the standard SLIC, IER, SLIC-GC [22] and Pol-IER) based on the simulated PolSAR data. The experiments demonstrate that the proposed method shows good performance with respect to the four criteria. In Section 4.2, experiments and discussions on two real-world PolSAR images are contained and the results show that the proposed method works best on boundary adherence and strong point targets preservation with higher computation efficiency compared with three state-of-the-art methods, i.e., the standard SLIC, IER and SLIC-GC. All the experiments were performed on a personal computer with 3.30 GHz Intel Pentium CPU, 4 GB memory and Matlab Code.

### 4.1. Evaluation on Simulated PolSAR Images

**Figure 2:**
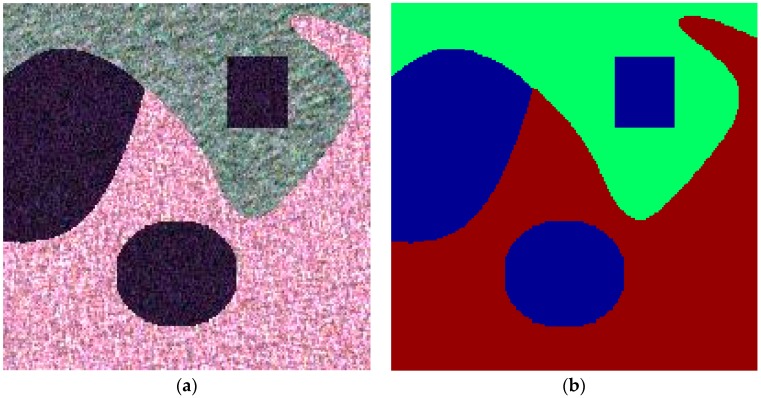
The simulated image with size 200×200 pixels and the corresponding ground truth. (**a**) Pauli_RGB image of the simulated Polarimetric synthetic aperture radar (PolSAR) data with three regular regions based on the inverse transform method and (**b**) the corresponding ground truth.

In our experiments, to evaluate the algorithms quantitatively, four simulated PolSAR images with three same regular regions but different sizes, i.e., 200×200 , 300×300 , 400×400 , and 500×500 pixels, were employed. The generation of simulated PolSAR images is based on the inverse transform method [31]. The simulated image with size 200×200 pixels is shown in Figure 2a and the corresponding ground truth is given in Figure 2b.

To evaluate the performance of different methods for superpixels’ generation, all the experiments on the four simulated PolSAR images mentioned above were assessed on four commonly used criteria: Boundary Recall (BR), Under-segmentation Error (USE), Achievable Segmentation Accuracy (ASA) and the running time. BR, USE and ASA are defined as follows:

1. Boundary Recall [32]: BR is the ratio of boundary pixels shared by the obtained superpixels and the ground truth, and it can be represented as
(19) $$\mathrm{BR}=N_{s\cap G}/N_{G}$$
  where $N_{s\cap G}$ denotes the number of superpixels’ boundary pixels overlapping the ground truth edges, and $N_{G}$ represents the number of ground truth edges. In this paper, the internal boundaries of the ground truth and superpixels were employed. So for BR, a larger value means better results.
2. Under-segmentation Error: if the ground truth segments are $g_{1},g_{2},\ldots ,g_{m}$ and the obtained superpixels are $s_{1},s_{2},\ldots ,s_{n}$ , the under-segmentation error is defined by [19,25]:
(20) $$USE=\frac{1}{N}\left[\sum_{i=1}^{M}\left(\sum_{\left[s_{j}|s_{j}\cap g_{i}>B\right]}\left|s_{j}\right|\right)-N\right]$$
  where N is the number of all the pixels in an image, $s_{j}\cap g_{i}$ is the overlapping error of the superpixel $s_{j}$ relative to a ground truth segment $g_{i}$ . |.| denotes the size of superpixels, and B represents a minimum number of pixels in $s_{j}$ overlapping $g_{i}$ . So USE should be as low as possible to obtain good superpixels.
3. Achievable Segmentation Accuracy [14]: ASA is a performance upperbound measure and the highest achievable accuracy of object segmentation when utilizing superpixels as units. By labeling each superpixel with the label of the ground truth segment that has the largest overlap, ASA can be computed as the fraction of correctly labeled pixels.
(21) $$ASA=\frac{\sum_{j}\max_{i}\left|s_{j}\cap g_{i}\right|}{\sum_{i}g_{i}}$$

#### 4.1.1. Evaluation on Two Distance Measures

In this paper, the fast revised Wishart distance instead of Wishart distance is employed as the data distance measure. Comparison experiments of our method with two distances were conducted on the four simulated PolSAR images to illustrate the superiority of the fast revised Wishart distance in our method.

**Figure 3:**
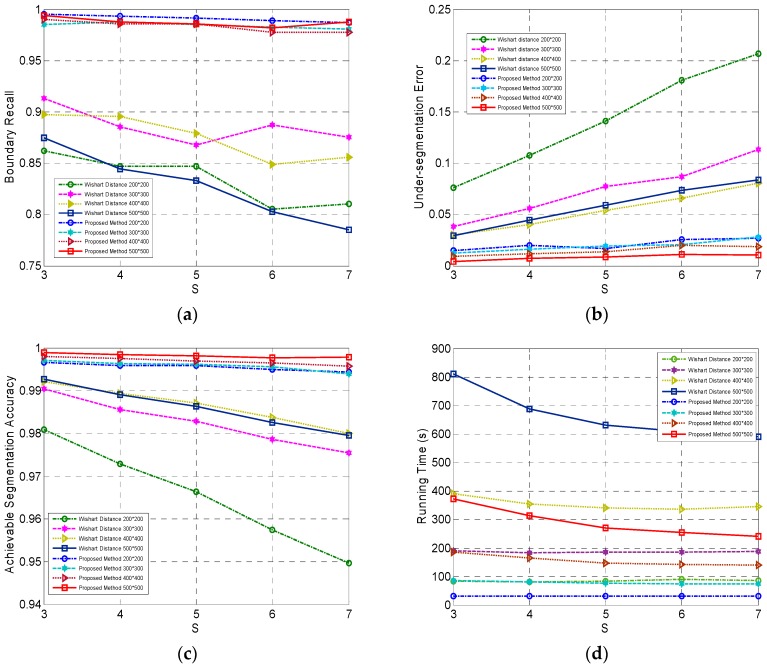
The comparison of our method with two different data distances, i.e., the fast revised Wishart distance and the Wishart distance with respect to four criteria of (**a**) Boundary Recall (BR); (**b**) Under-segmentation Error (USE); (**c**) Achievable Segmentation Accuracy (ASA) and (**d**) running time.

The compactness parameter m was set as 1.4 (the best) for the revised Wishart distance and 1 (the best) for the Wishart distance. The results on the four criteria are shown in Figure 3. We can find that the fast revised Wishart distance in our method was greatly superior to the Wishart distance in terms of all the four criteria. This is because the revised Wishart distance can better characterize the real data distance between coherency matrices than the Wishart distance and the fast implementation of the revised Wishart distance can significantly increase the computation efficiency. In addition, BR, USE, ASA and running time change very slightly with different initial grid width S , which is the expected property for superpixels. Therefore, the fast revised Wishart distance is chosen as the data distance measure in our proposed Pol-IER method.

#### 4.1.2. Evaluation of the Proposed Initialization Method

The initialization of unstable pixels is set as all the pixels instead of the grid edges scheme (GES) in IER. To demonstrate the advantage of the proposed initialization method, these two methods of initializing unstable pixels with the same steps were compared.

**Figure 4:**
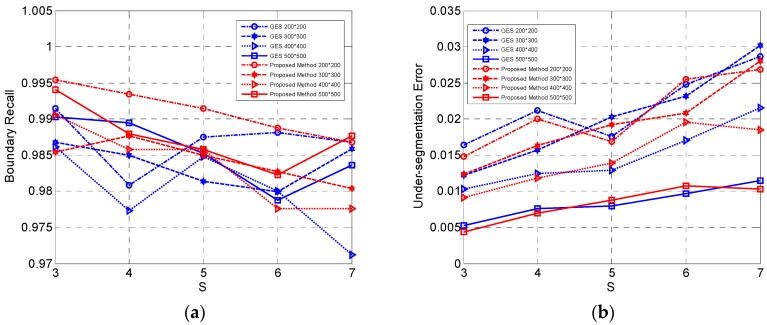

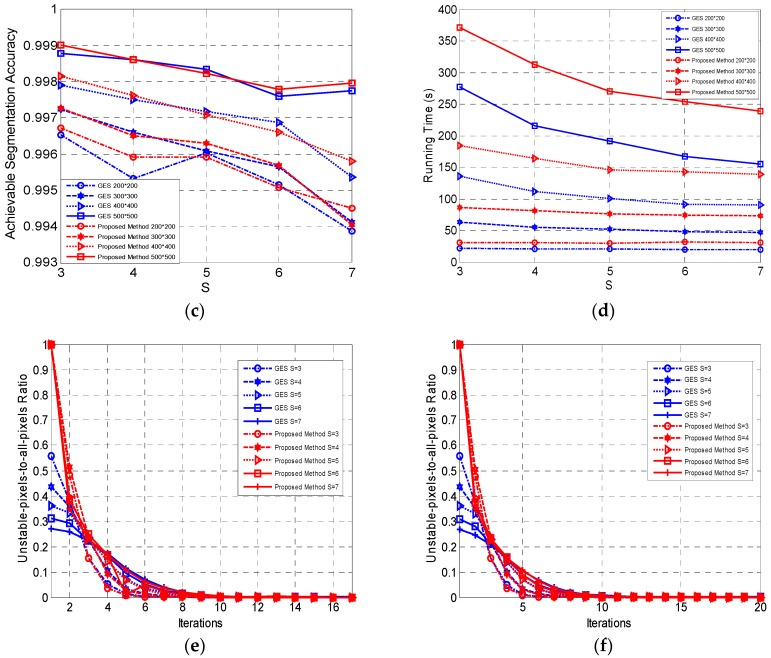
The comparison of two methods of initialization based on four simulated PolSAR images. (**a**) BR of two methods; (**b**) USE of two methods; (**c**) ASA of two methods; (**d**) running time of two methods; (**e**) the ratio of unstable pixels to all the pixels in the simulated PolSAR image with size 200×200 pixels; (**f**) the ratio of unstable pixels to all the pixels in the simulated PolSAR image with size 500×500 pixels.

The comparison results are shown in Figure 4. Figure 4a–c shows the slight advantage of our method in terms of BR, USE and ASA. That is because our method of initialization is proposed for those images with small-sized or slim regions which are common in real-world PolSAR images. The regions of simulated images are large and regular, thus leading to a small advantage for our initialization method. The superiority will be further shown in the experiments on the real-world PolSAR images in Section 4.2. The running time of our initialization with a larger number of initial unstable pixels is longer than the GES as shown in Figure 4d. The curves of unstable-pixels-to-all-the-pixels ratio with the iterations increasing are given in Figure 4e,f. Figure 4e,f is the results of the simulated PolSAR images with sizes 200×200 and 500×500 pixels, which are the smallest and largest size in all the represented simulated images, respectively. It can be seen that the ratio of the proposed method decreases more quickly than that of the GES and that the two methods will maintain a similar number of unstable pixels after three or four iterations. Therefore, the computation time is mainly spent on the first few iterations in Pol-IER, because the unstable pixels, of which most are the edges according to definition (1), are almost fixed in an image. Although our method takes a little longer, the curves change more slowly with different initial grid width compared with the GES, which means that the generated superpixels can maintain similar properties with different a grid width S . In addition, it can be seen from Figure 4d that the running time gap between our algorithm with the two initialization method will be widened with the size of PolSAR images increasing. This is mainly because when the size of the image is small, the initial unstable pixels to be relabeled in our method are of a small number, but with the large size of images to process, more pixels need to be relabeled by Pol-IER in the first few iterations. In summary, our proposed method of initialization outperforms the GES in terms of the expected properties with a little longer running time.

#### 4.1.3. Evaluation on Four Algorithms

Four algorithms including the standard SLIC, IER, SLIC-GC and Pol-IER were evaluated on the four simulated images. For these methods, a sequence of initial grid widths was set as {3,4,5,6,7} for wider comparison. The compactness parameter m was set as 15 for the standard SLIC and IER, 1.2 for SLIC-GC, and 1.4 for Pol-IER. Both $S_{\min}$ and $N_{th}$ were set as $S^{2}/4$ in the experiments.

**Figure 5:**
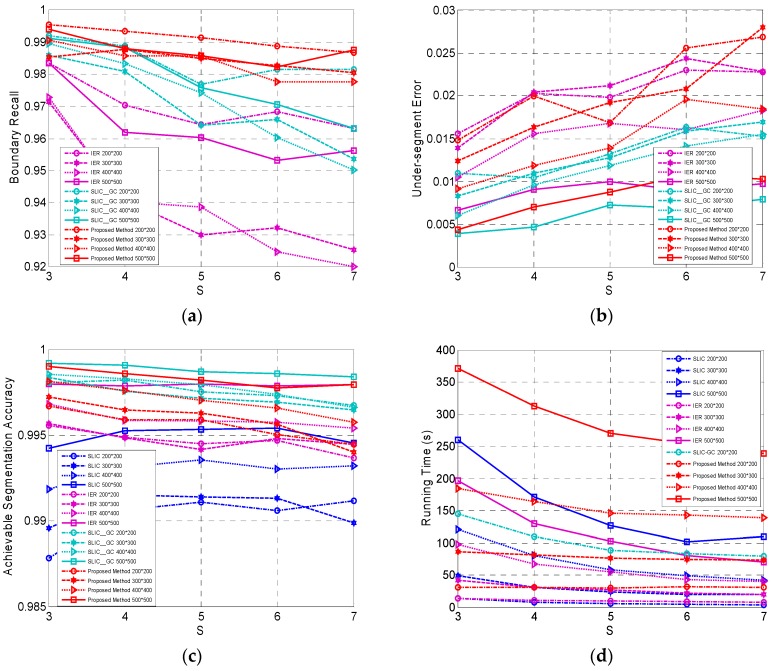
The results of four algorithms. (**a**) BR of IER, SLIC-GC [22] and Pol-IER; (**b**) USE of IER, SLIC-GC and Pol-IER; (**c**) ASA of the standard SLIC, IER, SLIC-GC and Pol-IER; (**d**) running time of the standard SLIC, IER, SLIC-GC and Pol-IER.

Figure 5 illustrates the results of the four algorithms on the four simulated images. The BRs of three methods, i.e., IER, SLIC-GC and Pol-IER are shown in Figure 5a, which does not include the BR of the standard SLIC for clarity because of the small value, about 10% lower than the other three algorithms. For BR, SLIC-GC outperforms IER, and Pol-IER is the best of the results, which indicates that the superpixels generated by Pol-IER have the boundaries closest to the real edges among the four methods. Figure 5b is the USEs of three algorithms similar to Figure 5a. The USE of the standard SLIC, which is the worst and thus not included in Figure 5b for clarity, is about 1.5% larger than those of the other three methods. IER is the second worst with a larger USE than SLIC-GC. As shown in Figure 5c, ASA has the same performance as the USE. Both the standard SLIC and IER utilize CIELAB Euclidean distance, which is unsuitable to describe the PolSAR data, thus leading to the worst BR, USE and ASA for them. Since the revised Wishart distance is employed in SLIC-GC and Pol-IER, SLIC-GC and Pol-IER obtain similar performance in terms of the three criteria, i.e., BR, USE and ASA. Although the USE and ASA of Pol-IER are generally slightly worse than that of SLIC-GC, Pol-IER, with the larger image size and small step, is comparable to SLIC-GC, whose running time is about one order of magnitude higher than that of Pol-IER with a large-sized image as shown in Figure 5d, where the running time curves of SLIC-GC on images with sizes 300×300 , 400×400 and 500×500 pixels are not included for clarity due to the values that are more than 10 times larger than other results . This is because the properties of superpixels generated by Pol-IER change slowly with different grid widths due to the initialization. With the same running time, Pol-IER can generate superpixels with a small S , which will lead to good performance, but IER can only employ a much larger S , with which poor superpixels will be yielded. Therefore, Pol-IER outperforms the others on overall performance.

### 4.2. Evaluation on Real-World PolSAR Images

**Figure 6:**
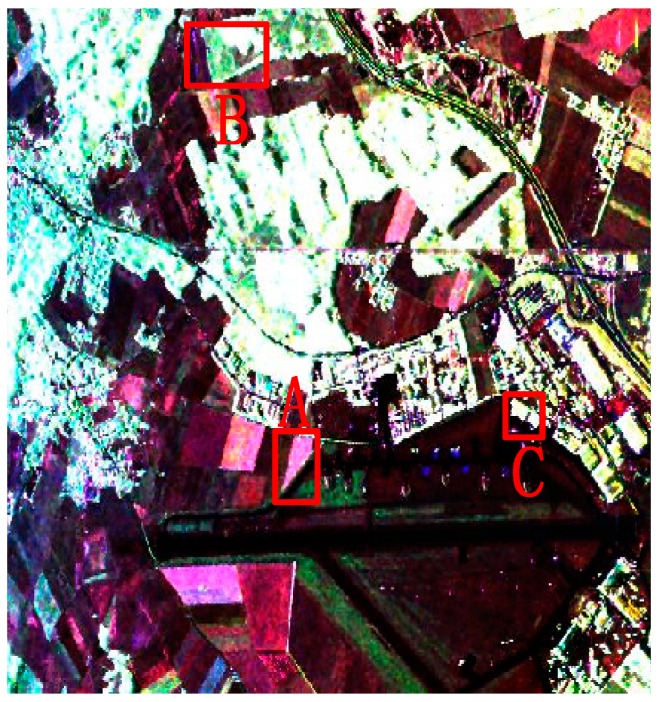
The Sinclair-RGB image of the real-world PolSAR image from Experimental Synthetic Aperture Radar (ESAR) with size 469 × 513. The patches A, B and C are enlarged in Figure 8.

To further evaluate the performance of Pol-IER, two real-world PolSAR images from ESAR and AirSAR were ultilized to conduct experiments. The first PolSAR data set is an L-band PolSAR image from the Oberpfaffenhofen test site, Oberpfaffenhofen, Germany. A preprocessing of six looks in the azimuth and three looks in the range were performed to equalize the resolutions of the azimuth and the range. After the multilook processing, the size is 469 × 513 pixels, and the Sinclair color-coded image is shown in Figure 6. The second data set is a 4-look AirSAR L-Band PolSAR image with size 750×1024 pixels from Flevoland, the Netherlands, and the Sinclair color-coded image is shown in Figure 11a.

The standard SLIC, IER, SLIC-GC and Pol-IER were performed on these two real-world images for comparison. The initial grid width is generally set according to the complexity of terrains empirically. In the experiments, all of the initial grid widths were set as S=5 for the first data set and S=12 for the second one. $S_{\min}$ was set as $S^{2}/4$ in CCA and $N_{th}$ was also set as $S^{2}/4$ for SLIC-GC and Pol-IER. The performance of these four competitive algorithms on real-world PolSAR images were visually inspected due to the absence of the ground truth of the real-world images. Therefore, we focused on the performance of the boundary adherence and strong point targets preservation for comparison.

#### 4.2.1. Evaluation on the First Data Set

**Figure 7:**
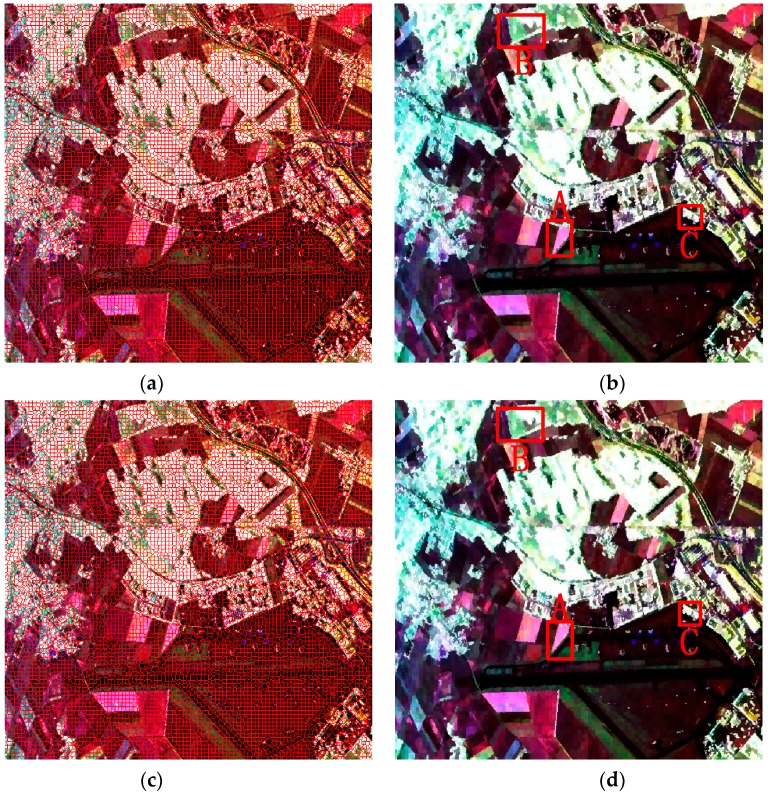
The generated superpixels of two methods of initialization. The first column denotes the final superpixel maps of different methods. The red lines superimposed onto the Sinclair-RGB images depict the superpixel boundaries. The second column gives the representation maps, where the coherency matrix of each pixel is replaced by the average value of the superpixel to which this pixel belongs. (**a**,**b**) are the generated superpixels of the method, which utilizes GES as the initialization step with m = 0.6. (**c**,**d**) are the generated superpixels of the proposed method with the same value of m.

**Figure 8:**
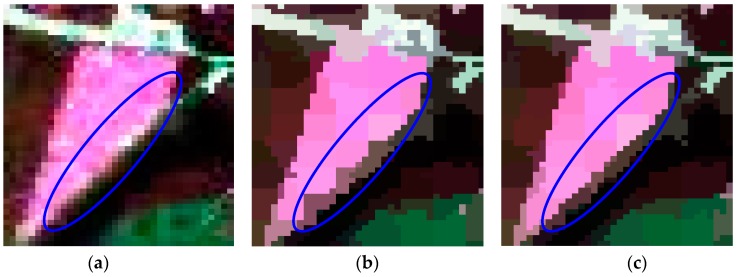

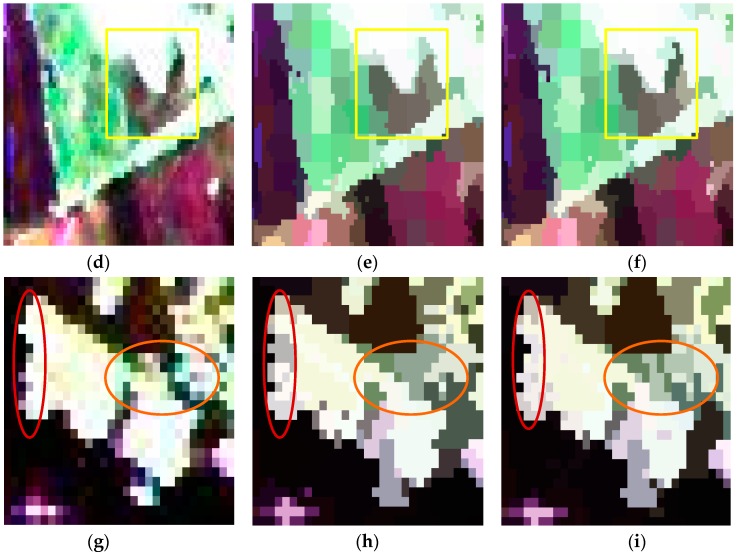
The details of regions A, B and C with two methods of initialization. The first column is the original images of three regions. The middle column is the results of the method with the grid edges scheme (GES). The last column denotes the results of our proposed method. (**a**,**d**,**g**) are the enlarged versions of areas A, B and C in Figure 6, respectively; (**b**,**c**) are the enlarged versions of areas A in Figure 7b,d, respectively; (**e**,**f**) are the enlarged versions of areas B in Figure 7b,d, respectively; (**h**,**i**) are the enlarged versions of areas C in Figure 7b,d, respectively.

Since the method of our proposed initialization, which initializes the unstable pixels as all the pixels in an image, only showed a slight advantage on the simulated PolSAR data for its large and regular regions, the comparison experiments of two methods of initialization were performed on the first real-world PolSAR image and the results are given in Figure 7. It can be seen that both methods work well as a whole. Since our method of initialization is proposed for the small or slim regions, we will analyze the details of the results. The details of regions A, B, and C, which are marked in red rectangles in Figure 7, are compared in Figure 8. It can be seen from the regions marked by blue ellipses in Figure 8b,c that our method has a smoother boundary than the GES. That is because our method of initializing unstable pixels contains all the potential edges, however, the GES may lose some edges, especially when the initial grid width is large. The region in the yellow rectangle of Figure 8f maintains a more similar shape in Figure 8d than that of Figure 8e, which also gives the credit to the proposed method of initialization. The last row in Figure 8 shows that more details are maintained by our method, which indicates that the proposed initialization method can preserve more detail information than the GES. In summary, our proposed method of initialization, compared with the GES, shows the superiority for PolSAR superpixel segmentation.

**Figure 9:**
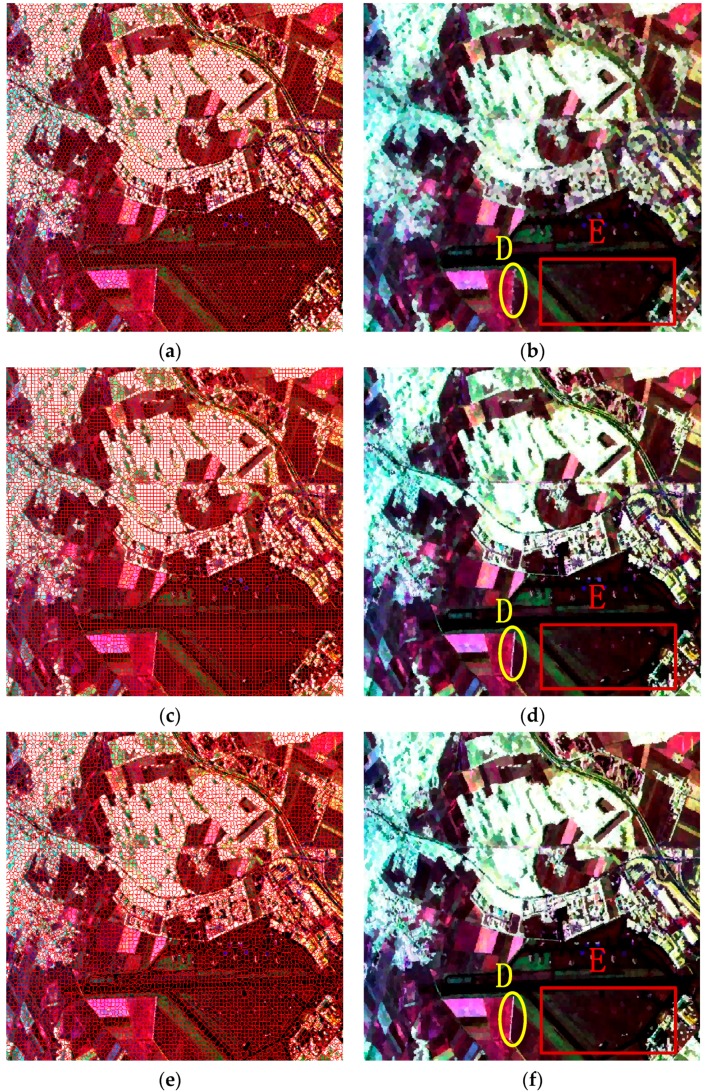

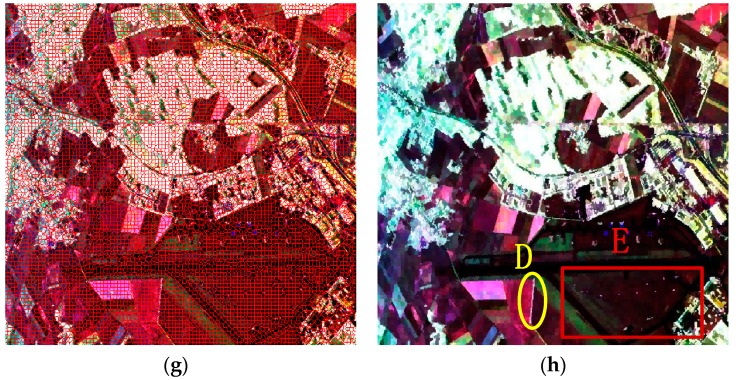
The generated superpixels of the four competitive methods. The first column denotes the final superpixel maps of different methods. The red lines superimposed onto the Sinclair-RGB images depict the superpixel boundaries. The second column gives the representation maps, where the coherency matrix of each pixel is replaced by the average value of the superpixel to which this pixel belongs. (**a**,**b**) are the results of the standard SLIC; (**c**,**d**) the results of IER; (**e**,**f**) the results of SLIC-GC and (**g**,**h**) the results of Pol-IER. The patches D and E in the second column images will be enlarged and compared in Figure 10.

Figure 9 gives the final superpixels of these four methods, in which the standard SLIC, IER, SLIC-GC and Pol-IER are included. The compactness m was set as 15 for the standard SLIC and IER, 0.3 for SLIC-GC and 0.6 for Pol-IER. It can be seen from Figure 9 that the standard SLIC acquired the worst superpixels. Although its running time, as shown in Table 1, is the shortest, the generated superpixels’ boundary of the standard SLIC cannot be preserved very well, especially in the slim regions and small regions such as the thin road in the middle of the image and the strong point targets in bottom right regions circled by the red rectangle. The reason is that all the pixels are relabeled according to CIELAB Euclidean distance, which makes more pixels incorrectly labeled. From the first column of Figure 9, it can be seen that the generated superpixels of IER and Pol-IER are regular in homogeneous areas with good boundary recall, because only unstable pixels rather than all the pixels are relabeled in the step of local relabeling in IER and Pol-IER.

**Figure 10:**
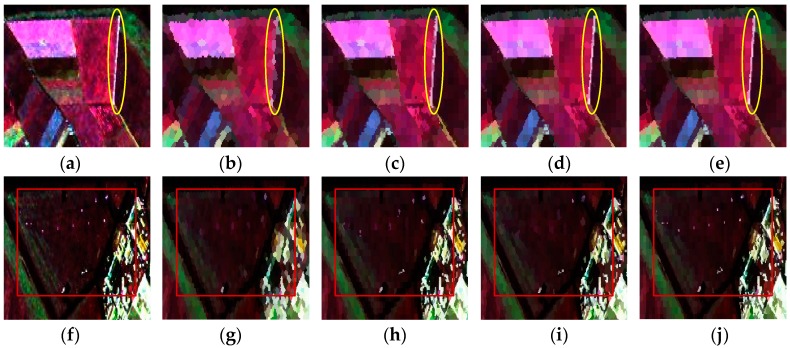
Two PolSAR image patches (first column) and corresponding superpixels provided by the standard SLIC (second column), IER (third column), SLIC-GC (fourth column) and Pol-IER (fifth column). (**a**,**f**) are the enlarged corresponding areas in Figure 6; (**b**–**e**) are the enlarged versions of areas A in Figure 9b,d,f,h, respectively; (**g**–**j**) are the enlarged versions of areas A in Figure 9b,d,f,h, respectively. More attention should be paid to the areas marked by squares and ellipses for comparison.

For visual clarity, the small regions D and E in Figure 9b,d,f,h are enlarged and shown in Figure 10. From the slim white regions marked by the yellow ellipses in Figure 10a–e, it can be seen clearly that both SLIC-GC and Pol-IER show good performance in boundary recall, because the revised Wishart distance can better describe the characteristic of PolSAR data than CIELAB Euclidean distance, which means that the coherency matrix provides more information than color for PolSAR images. From the regions marked by red rectangles (in Figure 10f–j), it can be observed clearly that the strong point targets can be preserved very well by Pol-IER because all the potential edges are maintained and the strong point targets are also preserved by the postprocessing procedure in Pol-IER, while most of the point targets are lost in the other three methods. In summary, Pol-IER performs the best in boundary adherence, regularity and strong point targets preservation.

The running time of these four methods are showed in Table 1. Since Pol-IER computed the distance based on the coherency matrix instead of the three elements in CIELAB space, the running time of Pol-IER is slightly longer than that of standard SLIC. Besides, only the grid edge pixels are considered as the unstable pixels for IER, while our Pol-IER takes all of the image pixels as unstable pixels in the initialization step, it is reasonable that our Pol-IER is less efficient than the IER. However, as a result of only relabeling unstable pixels, it can be seen that the running time of Pol-IER is more than eight times shorter than the state-of the-art SLIC-GC with the same data distance measure.

#### 4.2.2. Evaluation on the Second Data Set

**Figure 11:**
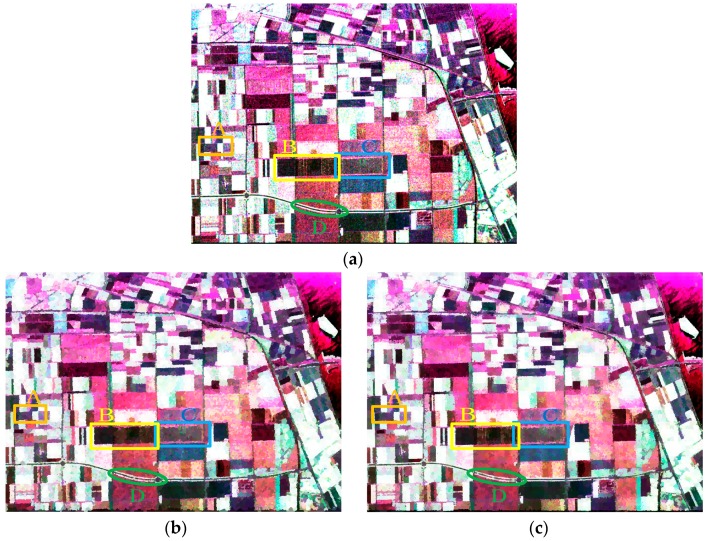

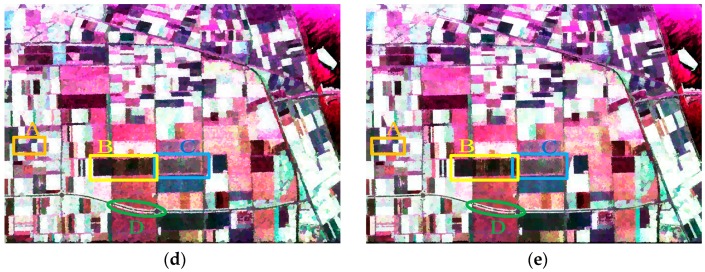
The results of four methods. (**a**) the Sinclair-RGB image; (**b**–**e**) give the representation maps of standard SLIC with m=18 , IER with m=18 , SLIC-GC with m=0.1 and Pol-IER with m=0.4 , respectively, where the coherency matrix of each pixel is replaced by the average value of the superpixel to which this pixel belongs. The patches A, B, C and D of (**a**–**e**) will be enlarged and compared in Figure 12.

**Figure 12:**
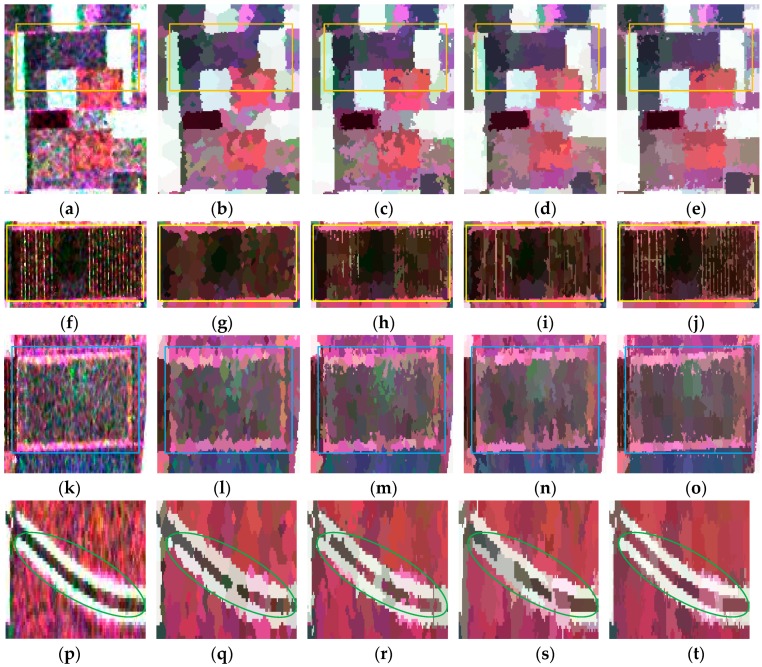
Four PolSAR image patches (first column) and corresponding superpixels provided by the standard SLIC (second column), IER (third column), SLIC-GC (fourth column) and Pol-IER (fifth column). (**a**–**e**) are the enlarged versions of areas A in Figure 11a–e, respectively; (**f**–**j**) are the enlarged versions of areas B in Figure 11a–e, respectively; (**k**–**o**) are the enlarged versions of areas C in Figure 11a–e, respectively; (**p**–**t**) are the enlarged versions of areas D in Figure 11a–e, respectively.

To further evaluate Pol-IER for real-world PolSAR images, experiments on the second data set were conducted. Figure 11 shows the results of the second PolSAR data set by the four algorithms. Figure 11b–e is the representation maps, where the coherency matrix of each pixel is replaced by the average value of the superpixel to which this pixel belongs. As a whole, it can be seen that the boundary of our method is smoother because of initializing unstable pixels as all the pixels. Figure 12 depicts the four patches, i.e., regions A, B, C and D in Figure 11a–e. Of all the boundaries in region A circled by the orange rectangles in Figure 12a–e, the last column (Figure 12e) has the smoothest boundary, which indicates that Pol-IER shows good performance with respect to boundary adherence. From region B in Figure 12f–j, it can be observed that the boundaries of the slim regions can be preserved best by Pol-IER due to the initialization step and the revised Wishart distance measure. The results of the region C in Figure 12k–o show that the boundaries generated by Pol-IER are smoother and closer to the real terrain edges. In Figure 12p–t, it can be seen that only the boundaries of the slim region marked by green ellipses generated by Pol-IER are preserved very well, which demonstrates the superiority of our method in boundary adherence. All the results in Figure 12 verify that Pol-IER shows good performance in boundary recall. One reason is that the revised Wishart distance can overcome the effect of speckle noise. Another reason is that initializing the unstable pixels as all the pixels and only relabeling unstable pixels can preserve more edges. In addition, the postprocessing procedure removes the small isolated regions as well as preserving strong point targets. Table 2 is the running time of these four methods. It can be seen that the running time of Pol-IER is about nine times shorter than that of SLIC-GC as a result of only relabeling unstable pixels.

In summary, the proposed method shows better performance than standard SLIC and IER, which is because the revised Wishart distance between two coherency matrices can better describe the data distance than the Euclidean distance in CIELAB color space, and the postprocessing based on a dissimilarity measure can better preserve the strong point targets than CCA. Our algorithm outperforms SLIC-GC as a result of initializing unstable pixels as all the pixels, which makes all the potential edges contained; only relabeling unstable pixels, which makes the proposed method more efficient and avoids incorrectly sorting more pixels; and implementing the revised Wishart distance in a fast way, which makes the computation efficiency further higher.

## 5. Conclusions

IER is a fast superpixel segmentation algorithm for optical images. Due to the inherent speckle noise and the existence of many small-sized or slim regions in PolSAR images, a fast PolSAR superpixel segmentation based on edge refinement and fast revised Wishart distance was proposed in this paper. The fast revised Wishart distance between two coherency matrices was utilized in the local relabeling of the unstable pixels instead of Euclidean distance in CIELAB color space to reduce the impact of speckle noise. To preserve the edges of the small-sized and slim regions as much as possible, the initialization of unstable pixels was set as all the pixels in PolSAR images rather than only the grid edge pixels. Finally, a postprocessing procedure, based on the dissimilarity measure, was employed to remove the generated small isolated regions as well as to preserve the strong point targets. The evaluations on four simulated PolSAR images with different sizes demonstrated the availability of the fast revised Wishart distance and the proposed initialization in terms of four commonly used criteria, i.e., BR, USE, ASA and running time. The experiments on two real-world PolSAR images showed that our proposed Pol-IER outperformed the other three methods including standard SLIC, IER and SLIC-GC in boundary adherence, regularity and strong point targets’ preservation. Although the running time is slightly longer than the standard SLIC and IER, it is much shorter compared with other methods of superpixel generation for PolSAR images and is even about nine times shorter than that of SLIC-GC with large-sized images. However, as a result of initializing all the image pixels as unstable pixels, the Pol-IER is more time consuming compared to the standard SLIC and IER which are originally proposed to generate superpixels for optical images. Therefore, it would be beneficial to further improve the efficiency of our proposed approach in future work.

## Figures and Tables

**Table 1:** The running time (in seconds) of four methods for the first real-world PolSAR image.

Algorithm	Clustering Time (s)	Postprocessing Time (s)	Total Time (s)
Standard SLIC	38.322	74.631	112.953
IER	64.518	26.127	90.645
SLIC-GC	2202.651	5.351	2208.002
Proposed Pol-IER	240.387	27.426	267.813

**Table 2:** The running time (in seconds) of four methods for the second real-world PolSAR image.

Algorithm	Clustering Time (s)	Postprocessing Time (s)	Total Time (s)
Standard SLIC	102.666	301.252	403.918
IER	326.867	52.447	379.314
SLIC-GC	5035.026	11.090	5046.116
Proposed Pol-IER	515.035	55.611	570.646
